# A comparison of manual neuronal reconstruction from biocytin histology or 2-photon imaging: morphometry and computer modeling

**DOI:** 10.3389/fnana.2014.00065

**Published:** 2014-07-11

**Authors:** Arne V. Blackman, Stefan Grabuschnig, Robert Legenstein, P. Jesper Sjöström

**Affiliations:** ^1^Department of Neuroscience, Physiology and Pharmacology, University College LondonLondon, UK; ^2^Institute for Theoretical Computer Science, Graz University of TechnologyGraz, Austria; ^3^Department of Neurology and Neurosurgery, Centre for Research in Neuroscience, The Research Institute of the McGill University Health Centre, Montreal General HospitalMontreal, QC, Canada

**Keywords:** morphology, reconstruction, cell-type classification, multicompartmental modeling, interneurons, 2-photon imaging, Neurolucida, neocortex

## Abstract

Accurate 3D reconstruction of neurons is vital for applications linking anatomy and physiology. Reconstructions are typically created using Neurolucida after biocytin histology (BH). An alternative inexpensive and fast method is to use freeware such as Neuromantic to reconstruct from fluorescence imaging (FI) stacks acquired using 2-photon laser-scanning microscopy during physiological recording. We compare these two methods with respect to morphometry, cell classification, and multicompartmental modeling in the NEURON simulation environment. Quantitative morphological analysis of the same cells reconstructed using both methods reveals that whilst biocytin reconstructions facilitate tracing of more distal collaterals, both methods are comparable in representing the overall morphology: automated clustering of reconstructions from both methods successfully separates neocortical basket cells from pyramidal cells but not BH from FI reconstructions. BH reconstructions suffer more from tissue shrinkage and compression artifacts than FI reconstructions do. FI reconstructions, on the other hand, consistently have larger process diameters. Consequently, significant differences in NEURON modeling of excitatory post-synaptic potential (EPSP) forward propagation are seen between the two methods, with FI reconstructions exhibiting smaller depolarizations. Simulated action potential backpropagation (bAP), however, is indistinguishable between reconstructions obtained with the two methods. In our hands, BH reconstructions are necessary for NEURON modeling and detailed morphological tracing, and thus remain state of the art, although they are more labor intensive, more expensive, and suffer from a higher failure rate due to the occasional poor outcome of histological processing. However, for a subset of anatomical applications such as cell type identification, FI reconstructions are superior, because of indistinguishable classification performance with greater ease of use, essentially 100% success rate, and lower cost.

## Introduction

Investigations of neuronal morphology have been a key feature of neuroscience since the studies of Ramón y Cajal and before (Ramón y Cajal, 1911; Senft, 2011). More recently, the drive to explain the relationship between neural structure and function has required more accurate and quantifiable models of neural morphology. Such reconstructions are vital across subfields such as cell-type identification (Ascoli et al., 2008), connectomics (Helmstaedter, 2013), computer modeling (Vetter et al., 2001; Sarid et al., 2007; Gidon and Segev, 2012) and studies of morphology itself (Cannon et al., 1999). Depending on the scope of the study, different levels of accuracy, completeness, resolution and throughput of reconstructions may be required; this is reflected in choice of imaging and reconstruction method, from electron microscopy to fluorescence imaging (FI). The development of techniques such as biocytin labeling of physiologically recorded cells, genetic labeling, 2-photon laser-scanning microscopy (2PLSM) and digital analysis have greatly aided efforts to bridge physiology and anatomy (Ascoli, 2006; Svoboda, 2011; Thomson and Armstrong, 2011). Detailed reconstructions, in combination with physiological data, have provided valuable insight into the connectivity, structure and function of neural circuits (Douglas and Martin, 2004). Increases in the number and accessibility of reconstructed neurons promise new approaches; for example, resources such as NeuroMorpho.Org allow researchers access to a large pool of reconstructions from published studies, which can be mined for further data (Ascoli et al., 2007). Use of such interlinked datasets of 3D reconstructions may be key in “big science” initiatives such as the Human Brain Project, and for any project wishing to simulate the brain (Markram, 2013).

Currently, digital reconstructions at the single-cell and microcircuit level are most often created manually using the Neurolucida system with biocytin labeled cells (Halavi et al., 2012). This said, neuronal reconstructions are increasingly based on other methods; for example fluorescent markers have been more frequently used over the past decade, and newer studies take advantage of technologies such as 2PLSM and freeware reconstruction software such as Neuromantic (Buchanan et al., 2012; Halavi et al., 2012; Myatt et al., 2012). However, the use of different reconstruction methods may yield different results. For example, BH based reconstructions can exhibit shrinkage and distortion when compared to reconstructions from 2PLSM FI (Egger et al., 2008). As such, the choice of reconstruction method could have a significant effect in itself on the results of e.g., cell classification and computer modeling. Despite this, there has been little quantification of the effects of method choice on morphological measurements and computer simulations. Here, we compare and contrast 16 reconstructions of the same 8 cells using the currently most popular method—Neurolucida reconstruction of biocytin-filled cells—and one increasing in use—reconstructions from 2PLSM FI stacks. We identify the strengths and weaknesses of either method for specific applications, and we make recommendations as to their appropriate use.

## Methods

### Electrophysiology/slice preparation

Procedures conformed to the *UK Animals (Scientific Procedures) Act 1986* and to the standards and guidelines set in place by *the Canadian Council on Animal Care*, with appropriate licenses. Mice aged P12-P20 were anesthetized with isoflurane and decapitated. Brain dissection was performed in ice-cold artificial cerebrospinal fluid (aCSF; in mM: NaCl, 125, KCl, 2.5; MgCl_2_, 1; NaH_2_PO_4_, 1.25; CaCl_2_, 2; NaHCO_3_, 26; Dextrose, 25; bubbled with 95% O_2_/5% CO_2_). Acute brain slices (visual cortex, near-coronal, 300 μm thick) were prepared with a Leica VT1200S vibratome, and incubated in 37°C aCSF for up to 1 h, after which they were allowed to cool to room temperature. Patch-clamp recordings were then performed in slices in the whole-cell configuration at 32-34°C. Patch pipettes (4–6 MΩ) were produced with a P-1000 electrode puller (Sutter Instruments) from medium-wall capillaries, and held internal solution containing, in mM: KCl, 5; K-Gluconate, 115; K-HEPES, 10; MgATP, 4; NaGTP, 0.3; Na-Phosphocreatine, 10; for imaging/reconstruction: 10–40 μM Alexa Fluor 594 and 0.5–1.0% w/v Biocytin. Internal was adjusted with KOH to pH 7.2–7.4. Primary visual cortex was targeted based on the presence of a granular layer 4. All recordings were performed in layer 5 (L5), identified by the presence of large L5 pyramidal cell (PC) somata. L5 PCs were targeted based on a thick apical dendrite; interneurons (INs) were targeted based on small, rounded somata, and were verified by fast-spiking response to rheobase current injection. PCI-6229 boards (National Instruments, Austin, TX) were used for data acquisition, with custom software (Sjöström et al., 2001) running in Igor Pro 6 (WaveMetrics Inc., Lake Oswego, OR). All recordings were made in current clamp and were filtered at 5–6 kHz and acquired at 10 kHz. Neurons were patched at 400X or 600X magnifications using a SliceScope (see below, Scientifica Ltd.) with infrared video Dodt contrast. All recordings were made in the C57BL/6 strain. Electrophysiology procedures were used solely to ascertain cell health, fill cells with dyes and verify cell-type online by inspection of spiking properties.

### Histological processing and neurolucida reconstruction

After recording, slices were histologically processed to enable biocytin-based reconstructions. Slices were fixed in 4% paraformaldehyde/4% sucrose in phosphate-buffered saline (PBS; pH 7.2–7.4) overnight at 4°C. The following day, slices were washed for 3 × 15 mins in PBS. Subsequently, slices were permeabilized in pre-cooled 100% methanol at −20°C for 5–10 mins. Slices were then washed in PBS a further 3 × 10 mins. Endogenous peroxidases were blocked in 1% H_2_O_2_ for 15 mins at room temp. Further 3 × 5 min PBS washes were performed. Slices were then incubated with Vectastain ABC elite kit (Vector Labs) overnight at 4°C. The next day, slices were washed a further 3 × 10 mins in PBS, and incubated with ImmPact SG Peroxidase substrate (Vector Labs) to initiate staining reaction. The staining was stopped when developed (around 10 mins) with PBS. Further 3 × 5 min PBS washes were performed, and slices were mounted/coverslipped in Mowiol (Sigma-Aldrich). Filled neurons in mounted and coverslipped slices were reconstructed using the Neurolucida system (MBF Bioscience) with a 100× oil-immersion objective. Resulting Neurolucida DAT files were converted to SWC using the freeware NLMorphologyConverter (www.neuronland.org).

### 2-photon imaging and fluorescence reconstruction

2PLSM (Denk et al., 1990) was performed using a workstation custom built from a SliceScope (Scientifica) microscope fitted with an MDU (Scientifica), with photomultipliers in epifluorescence configuration. Scanners were Thorlabs GVSM002/M 5-mm galvanometric mirrors. A MaiTai BB (Spectraphysics) Ti:Sa laser tuned to 800–820 nm for Alexa 594 excitation was used for excitation. Uniblitz LS6ZM2/VCM-D1 shutters were used to gate the laser, while laser power level was controlled manually using a polarizing beam splitter (Melles Griot PBSH-450-1300-100 with AHWP05M-980 half-wave plate) and monitored using a power meter (Melles Griot 13PEM001/J) after a fraction of the beam was picked off with a glass slide.

PCI-6110 boards (National Instruments) were used to acquire imaging data using custom versions of ScanImage v3.5–3.7 (Pologruto et al., 2003) in Matlab (MathWorks, Natick, MA). 3D image stacks with slices of 512 × 512 pixels were acquired at 2 ms/line with z-steps of 1–2 μm. To reduce noise, each slice of the stack was an average of three frames. Resulting TIFF stacks were subsequently 3D-median filtered for inspection and for figures, but not for the reconstruction process. Stack brightness and contrast were altered in MacBiophotonics ImageJ (www.macbiophotonics.ca). Parameters were chosen to allow visualization and manual tracing of neurites with the least possible artificial enlargement of diameters. Registration of stacks was performed manually in Neuromantic (http://www.reading.ac.uk/neuromantic) and reconstruction of neurons was performed in this environment.

### Morphological analysis

Images of reconstructed cells (e.g., **Figure 2**) were rendered using NEURON. Quantitative analysis of reconstructions in SWC format was performed using either L-measure (Scorcioni et al., 2008), for which details of each function are available at http://cng.gmu.edu:8080/Lm/help/index.htm, or with our custom software qMorph written in Igor Pro, previously described in Buchanan et al. (2012). In L-measure, results are for the entire cell (axons and dendrites pooled together). The L-measure function “Length” refers to average compartment length, so in Table 1 we have referred to this as “Compartment length” for clarity. Custom software was used to create density maps, convex hulls and Sholl analysis (Sholl, 1953). Prior to analysis, morphologies were rotated slightly (16.97 ± 5.36° on average) to align apical dendrite/pial surface directly upward. Morphologies were aligned on the soma for all analyses.

**Table 1:**
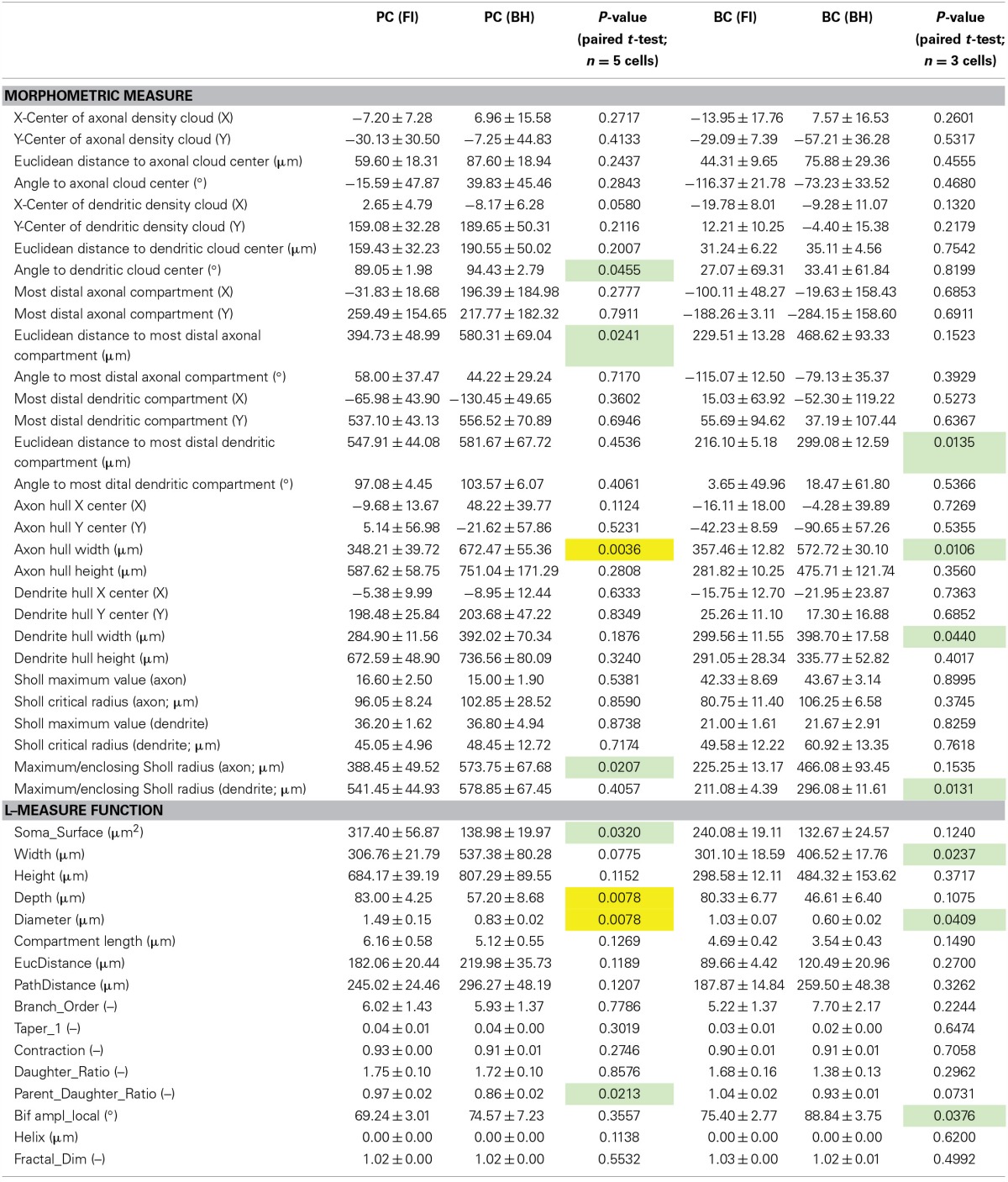
**Morphometry**.

*Morphological measures used for comparison of reconstruction methods (**Figures 2, 3**) in pyramidal and basket cells. Measures were generated using either in-house software (see Methods) or L-measure (listed as function names from the software to reduce ambiguity). Comparisons with significance levels p < 0.05 and p < 0.01 are highlighted in green and yellow respectively. (–) indicates unit less measures such as counts and ratios*.

To create density maps, each compartment of a reconstruction was represented by a 2D Gaussian aligned on its XY center, with its amplitude proportional to compartment length and its sigma fixed to 25 μm. These Gaussians were summed to create a smoothed 2D projection of morphology (density map). Axon and dendrite were treated separately. Individual density maps were peak normalized to enable averaging across reconstructions. Symmetry in density maps is a result of mirroring of reconstructions, however analyses on individual cells were performed on non-mirrored data. Ensemble maps for axon and dendrite were normalized, assigned color lookup tables and merged with a logical OR (e.g., **Figure 2**). Gamma correction was used to better visualize weak densities.

Convex hulls were created for each reconstruction based on 2D projections of axonal and dendritic arbors, using the gift-wrapping algorithm, also known as the Jarvis march (Jarvis, 1973). Ensemble hulls are convex hulls of all hulls of a certain type, including mirror images. Sholl analysis was performed in radial coordinates, moving in increasing 6.5 μm steps from *r* = 0, with the origin centered on the cell soma, and counting the number of compartments crossing a given radius. Sholl diagrams are averaged without normalization. Maximum value is the maximum number of crossings, whilst critical radius is the radius at which the maximum number of crossings was found. Maximum Sholl radius is the furthest radius with at least one crossing (the enclosing radius).

Process diameters were calculated using L-measure to obtain averages of cells (axon and dendrite measured separately). Diameters of visually matched locations between reconstructions of the same cells with different methods were measured manually in Neuromantic.

### Statistical comparisons

Results are reported as mean ± s.e.m. unless otherwise stated. Comparisons were made using paired samples *t*-test for equal means, unless otherwise stated. No corrections for multiple comparisons were applied, as for the purposes of this paper we feel it is more important and preferable to highlight potential differences between methods than to overlook them. Statistical tests were carried out in Igor Pro, Microsoft Excel and/or JMP (SAS). At least three animals were used for each group analyzed, and *n*_cell_ = *n*_animal_ (Aarts et al., 2014). Significance levels *p* < 0.05, *p* < 0.01 and *p* < 0.001 are denoted by one, two, and three stars respectively.

### Data clustering

Multidimensional hierarchical data clustering was performed on the first two principal components of standardized data in JMP using Ward's method and the Euclidean distance as linkage metric; or normal mixtures iterative clustering, which is based on the expectation-maximization algorithm (http://www.jmp.com/support/help/Normal_Mixtures.shtml). Prior to clustering, we performed principal component analysis on all variables listed in Table 1. In order to achieve fair weighting of morphological features in clustering, we identified pairs of variables in the resulting correlation matrix where *r* > 0.8, and excluded the variable which had the lower loading value in PCA (Tsiola et al., 2003). Clustering of morphologies was thus performed on the first 2 principal components of 27 measured parameters. From L-measure, we used Diameter, Length, PathDistance, Branch_Order, Taper_1, Contraction, Daughter_Ratio, Parent_Daughter_Ratio, Partition_asymmetry, Bif_ampl_local, Helix, Fractal_Dim. From our custom software qMorph, we used distance to center of axonal cloud, angle to center of axonal cloud, most distal axonal compartment x-coordinate, most distal axonal compartment y-coordinate, most distal dendritic compartment x-coordinate, angle to most distal dendritic compartment, axon hull x-center, axon hull width, dendritic hull x-center, dendritic hull y-center, dendritic hull width, axon Sholl max value, axon Sholl critical radius, dendrite Sholl critical radius, axon Sholl maximum/enclosing radius.

### Simulations

All Simulations were performed in NEURON 7.2 (Hines and Carnevale, 1997). Plots were created using a combination of Matlab and Igor Pro.

To explore the differences in the electrical behavior of FI and BH reconstructions of the same original cell, we studied active back propagation of APs and passive forward propagation of EPSPs along the apical dendrite of NEURON models based on these reconstructions. During a simulation, the peak potential at every segment along a path from the soma to the apical tuft was recorded and was plotted against the distance of the recording site from the origination point of the apical dendrite. The distance was measured as the Euclidean distance between the two points in space, and a path from soma to the tip was picked by hand.

#### Model initialization

In order to build a model from the reconstructions, the active and passive membrane properties from the model of Stuart and Häusser (2001) were used. The passive membrane properties were initialized with specific membrane and axial resistivities R_M_ of 12,000 Ωcm^2^, R_A_ of 150 Ω cm and a specific membrane capacitance C_M_ of 1 $\frac{\mu F}{cm^{2}}$. Active membrane conductances constituted by mechanisms for fast sodium and slow potassium currents were uniformly distributed over the membrane with ${\bar{g}}_{\text{Na}}=30\;\frac{pS}{\mu m^{2}}$ and ${\bar{g}}_{\text{Kv}}=50\frac{pS}{\mu m^{2}}$ in dendrites and at the soma. To avoid end-effects the sodium conductance in basal dendrites and apical oblique dendrites was reduced to ${\bar{g}}_{\text{Na}}=8\frac{pS}{\mu m^{2}}$. In dendrites, all conductances and the capacitance were multiplied by 2 to account for spines. The axon was treated as completely myelinated without spike initiating regions with ${\bar{g}}_{\text{Na}}=10\frac{pS}{\mu m^{2}}$, and ${\bar{g}}_{\text{Kv}}=0\frac{pS}{\mu m^{2}}$ and a reduced C_M_ of 0.04 $\frac{\mu F}{cm^{2}}$.

#### Backpropagation of APs

To standardize across reconstructions, a rheobase spike was generated and recorded. All backpropagation simulations were performed by replaying this spike at the soma. For spike generation, a spike-initiating hillock was added to the reconstruction PC FI 2 (20130205) with ${\bar{g}}_{\text{Na}}=10000\frac{pS}{\mu m^{2}}$ and ${\bar{g}}_{\text{Kv}}=500\frac{pS}{\mu m^{2}}$. The rheobase spike was then triggered by injection of a 5 ms current of 1.0215 nA.

#### Forward propagation of EPSPs

For EPSP generation, an alpha-synapse with a τ_rise_ of 0.3 ms, a τ_fall_ of 3 ms and a g_max_ of 5 nS was used. This was inserted at a dendritic location with prominent surrounding morphology, to ensure that it could reliably be positioned at an identical location for both the BH and the FI reconstructions of the same neuron.

#### Length constants

Length constants were determined by injecting a 300-ms-long constant current of 50 pA at matched locations (as with the EPSPs above). When steady state was reached (we arbitrarily picked *t* = 149 ms), the membrane voltage was plotted vs. distance from injection site. Length constants λ, were measured by fitting exponentials to these plots in Igor PRO.

## Results

### Morphometric comparison of reconstruction methods

**Figure 1:**
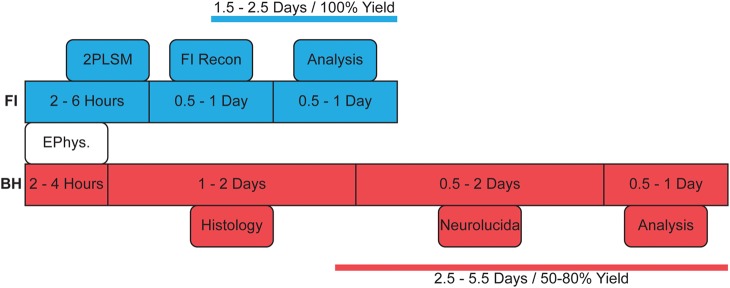
**Flowchart indicating typical reconstruction steps with either method**. BH reconstructions take longer due to histology and require multiple setups for recording and reconstruction with Neurolucida. As FI can be monitored online during 2PLSM image acquisition, there is in effect a 100% yield of complete reconstructions, whereas with BH, histological processing occasionally fails or is incomplete, in our hands giving a yield of around 50–80% (see main text).

**Figure 2:**
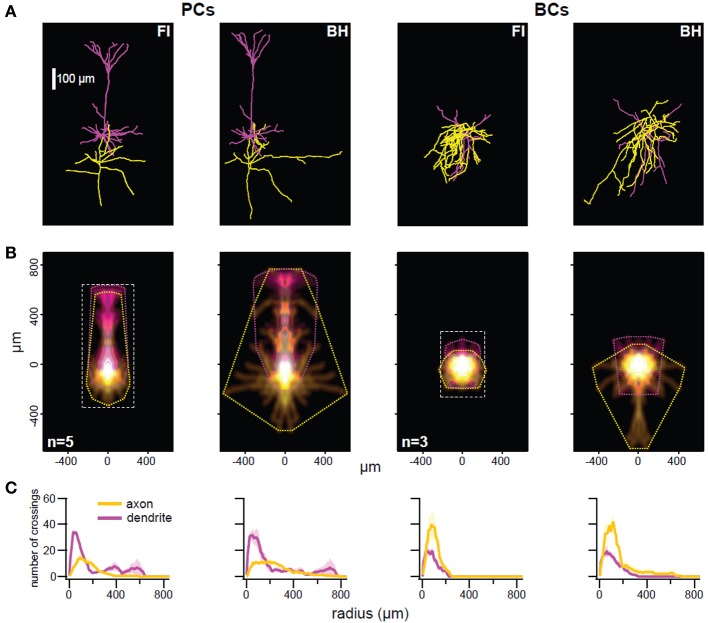
**The BH approach enables better reconstruction of thin distal arbors. (A)** Representative reconstructed morphology pairs of a single pyramidal cell (PC; left) and basket cell (BC; right) generated with fluorescence imaging (FI) or biocytin histology (BH). Reconstructions appeared qualitatively similar, but BH allowed for tracing of longer collaterals. There was also some expansion of BH reconstructions in XY, perhaps due to compression (see main text). **(B)** Density maps indicate average distribution of axonal (yellow) and dendritic (magenta) arbors, whilst convex hulls (dotted lines) show maximum extent. Reconstructions are aligned on soma. For FI reconstructions, the imaged area is represented by a dotted rectangle, outside of which any arbors would have been missed. Axonal convex hull width was larger in BH reconstructions for PCs (*p* < 0.01) and BCs (*p* < 0.01), as was dendritic hull width for BCs (*p* < 0.05). Distance to furthest axonal compartment was larger for BH reconstructions of PCs (*p* < 0.05), whilst distance to furthest dendritic compartment was larger for BH reconstructions of BCs (*p* < 0.05). Angle to relative dendritic center was larger in BH PC reconstructions (*p* < 0.05). Other measures were not significant. See Table 1 and Methods for full details. **(C)** Sholl analysis (see Methods) of each cell type/reconstruction method. Maximum value and critical radius were not significant for any comparison, however furthest radius with at least one crossing was significantly larger in BH reconstructions of PCs for axon (*p* < 0.05) and in BH reconstructions of BCs for dendrite (*p* < 0.05). Yellow and magenta denote axon and dendrite crossings, with paler hues indicating ±SEM. See Table 1 for details.

Neocortical L5 pyramidal cells (PCs) and basket cells (BCs) were targeted based on soma shape and were subsequently identified by spiking properties (data not shown) and morphology. We filled cells with both biocytin and Alexa 594, and reconstructed using Neurolucida software on BH tissue and Neuromantic software on 2PLSM FI stacks, resulting in two morphological reconstructions of each cell (see Methods and Figure 1). Subjectively, reconstructions appeared similar with both methods, although BH allowed tracing of horizontal axonal/dendritic collaterals for longer distances (Figure 2A), perhaps because thin distal processes dye-filled so slowly that BH but not FI distal tips were readily visualized. In addition, BH involves an amplification step that further improves visualization of poorly labeled processes. PCs were identified by their characteristic apical dendrite, and their axons were largely confined to L5 with the occasional ascending process. BCs were characterized by axonal and dendritic arbors ramifying extensively within L5, with few processes venturing outside this layer.

We quantitatively analyzed morphology with L-measure, a freely available software for morphological analysis (Scorcioni et al., 2008). Comparison of measurements for entire cells (see Table 1) revealed a wider arbor width for BH reconstructions of BCs (*p* < 0.05), and smaller depth (*p* < 0.01) and somatic surface area (*p* < 0.05) for BH reconstructions of PCs (Table 1). Whilst a wider arbor width for BH BC reconstructions likely reflects the greater ease of tracing distal collaterals with this method, the smaller depth and somatic surface area of BH PC reconstructions are likely due to shrinkage during fixation and differences in software soma modeling, respectively.

Examination of branch-level and bifurcation-level measures (Table 1, see Methods), using L-measure highlighted the general similarity of reconstructions, as most metrics were indistinguishable (Table 1). That said, parent-daughter ratio, defined as the ratio of process diameter between daughter and parent at each bifurcation point, was significantly lower for BH PC reconstructions (*p* < 0.05). Local bifurcation amplitude (angle between two new branches at a bifurcation) was also significantly larger for BH BC reconstructions (*p* < 0.05; Table 1).

When quantifying morphology, it is often useful to separately analyze axonal and dendritic segments. For example, axonal morphology is thought to be more important than dendritic morphology for IN classification (Markram et al., 2004; Ascoli et al., 2008; DeFelipe et al., 2013). As previously described (Buchanan et al., 2012), we also analyzed morphology by comparison of axonal and dendritic convex hulls and density maps using custom software (Figure 2B; Table 1; see Methods). Whilst reconstruction with BH allowed tracing of more distal collaterals, reflected by significant differences in mean axon hull width (*p* < 0.01) and distance from soma to the furthest axonal compartment (*p* < 0.05) for PCs, and both axonal (*p* < 0.05) and dendritic (*p* < 0.05) hull width and distance from soma to the furthest dendritic compartment (*p* < 0.05) for BCs, most other measures derived this way were indistinguishable between reconstruction methods (for full detail see Table 1). This suggests that FI and BH may perform similarly for cell classification and morphometry that does not rely chiefly on thin distal tips of arborizations. In addition, indistinguishable measures included the relative density and hull centers of axonal and dendritic arbors, indicating that both methods are in fact comparable in revealing the majority of axonal and dendritic morphology.

Angle to the center of the dendritic density cloud was significantly but only slightly different between FI and BH reconstructions for PCs (*p* < 0.05; Table 1), but not for BCs. Although significant, this may be a spurious finding, since reconstructions were manually aligned to point straight up, which may introduce human error and a bias. However, this remained significant even when we tried to carefully account for any bias, so we report this as is.

Sholl analysis (Sholl, 1953) is a classical quantitative method used to analyze neuronal morphology based upon the number of crossings made by processes over usually soma-centered concentric circles of increasing radius. Sholl analysis indicated that both methods yielded largely similar reconstructions (Figure 2C); differences in maximum value and critical radius (see Methods) were not significant for either cell type (Table 1). However, the furthest radius with at least one crossing was larger with BH for axon but not dendrite in PCs, and dendrite but not axon for BCs (Table 1). This probably reflects both the capacity to visualize more distal processes with BH, and shrinkage or compression of BH-processed slices after coverslipping. Compression results in smaller depth of BH reconstructions and to expansion in the XY axes (see Table 1).

Overall, whilst BH allows better reconstruction of very distal processes, seen in e.g., wider arbor extents and maximum Sholl radii, reconstructions were largely indistinguishable between methods (Table 1), indicating that both methods are suitable for analysis of morphology. Although FI/2PLSM based reconstructions are limited by the extent of imaging captured, it may be possible to recover more distal processes using this method by capturing images from a wider area, even if there does not appear to be fluorescence signal when viewing online (see area imaged for FI reconstructions, Figure 2B).

**Figure 3:**
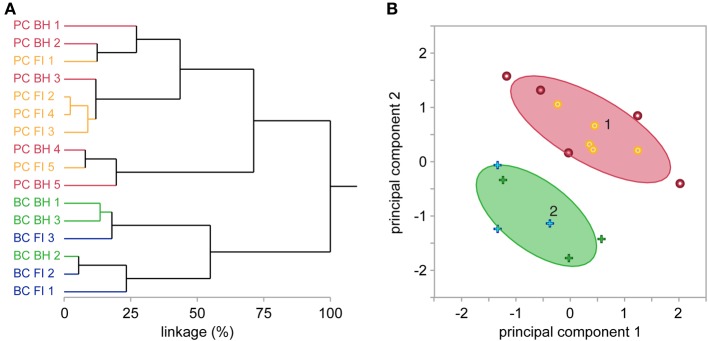
**BH and FI reconstruction methods have similar overall morphometric performance. (A)** Hierarchical clustering of the first 2 principal components of 27 morphological variables (see Methods) independently segregated all reconstructed cells into two major clusters, each exclusively containing PCs or BCs. Further subclusters did not segregate reconstructions from FI or BH. Taken together, this indicates their similarity for morphological cell classification. Each label on the y-axis is a reconstruction, with coloring indicating cell and method type. Linkage distance is plotted on the x-axis, indicating the level of dissimilarity between clusters. **(B)** In agreement, expectation-maximization clustering also separated BCs from PCs. Crosses denote BCs, and dots PCs. As in **(A)**, coloring indicates reconstruction method (blue or yellow = FI; green or red = BH). Ovals denote the region where 90% of observations in each cluster are expected to fall.

When investigating neural circuits, it is vital to properly identify anatomical cell type as, for example, synaptic features may differ widely at connections between different cells (Ascoli et al., 2008; Blackman et al., 2013; DeFelipe et al., 2013). We explored the impact of reconstruction method on cell classification using multidimensional hierarchical clustering of all reconstructions from both methods (see Methods and Figures 3A,B). This approach independently segregated reconstructions into two major clusters, each containing exclusively BCs or PCs. Within the two BC and PC clusters, however, reconstructions from BH or FI did not further segregate into distinct sub-clusters. Taken together, these results suggest that both reconstruction methods produce enough detail to reliably classify different neuronal types, while at the same being so similar in terms of outcome that the choice of method does not impact cell classification appreciably. This said, a pair of reconstructions of the same cell using BH and FI formed a nearest-linkage neighbor in only one case (BC 2; Figure 3A), highlighting that whilst classification performance was similar between methods, there were still appreciable morphological differences between reconstructions of the same cell completed with BH or FI. Clustering of all reconstructions into two groups using the expectation-maximization algorithm (normal mixtures clustering in JMP) also separated PCs and BCs with no errors (Figure 3B). Whilst clustering of morphologies resulted in two major cell classes here, it should be noted that both PCs (Groh et al., 2010) and BCs (Markram et al., 2004) may consist of further subtypes.

### Reconstructions from 2PLSM have larger process diameter

**Figure 4:**
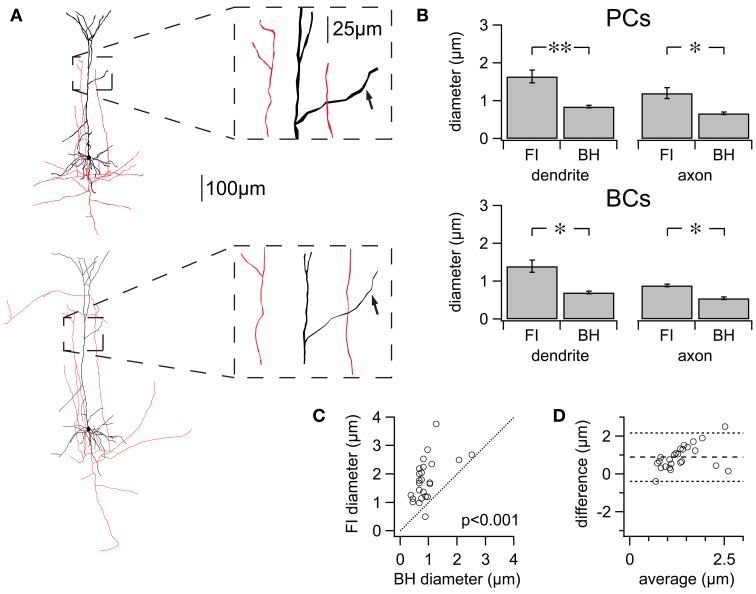
**FI reconstructions suffer from systematically enlarged process diameters. (A)** Two reconstructions of the same cell using FI (top) and BH (bottom). Inset: zoom highlighting differences in diameter for dendrite (black) and axon (red). Arrows in inset: example of matched dendritic locations, quantified in Figures 3C,D. **(B)** Average differences in process diameter for PCs and BCs using either method. FI reconstruction resulted in consistently larger diameter for PCs (*n* = 5 cell pairs, FI vs. BH; axon 1.20 ± 0.14 μm vs. 0.67 ± 0.04 μm, *p* < 0.05; dendrite 1.65 ± 0.17 μm vs. 0.84 ± 0.03 μm, *p* < 0.01) and BCs (*n* = 3 cell pairs; axon 0.89 ± 0.04 μm vs. 0.55 ± 0.04 μm, *p* < 0.05; dendrite 1.40 ± 0.16 μm vs. 0.71 ± 0.03 μm, *p* < 0.05). Average diameters for entire cells are found in Table 1. **(C)** Differences in diameter for manually matched dendritic locations using either method (see Figure 3A). All but one matched measurements were plotted above the line of equality, reflecting the tendency of FI reconstructions to have larger process diameter (PCs; *n* = 5 cell pairs; *n* = 25 segment pairs; FI vs. BH Diameter; mean 1.80 ± 0.15 μm vs. 0.91 ± 0.09 μm; *p* < 0.001). **(D)** The degree of agreement between the two methods is ascertained using a Bland-Altman or Tukey mean-difference plot (Bland and Altman, 1986). FI diameter—BH diameter is plotted against averaged process diameters, (FI+BH diameter)/2. Middle dotted line indicates a positive mean difference (0.89 ± 0.13 μm), showing that FI reconstructions consistently suffer from exaggerated process diameters. The upper and lower dotted lines indicate ±2SD and the 95% limits of agreement (*SD* = 0.64 μm). Linear regression (not shown) identified a significant slope (0.56; *p* < 0.05), showing that FI reconstruction overestimates diameters more for larger diameters. ^*^p < 0.05, ^**^p < 0.01.

When creating 3D reconstructions of neurons to be used for e.g., computer modeling, it is important for these to be as accurate as possible, as even quite subtle structural differences can have quite dramatic effects on biophysical properties (Vetter et al., 2001; Schaefer et al., 2003). For example, differences in process diameter between reconstructions will affect membrane surface area, process volume, number of ion channels, axial resistance, length constant, and in turn propagation of electrical signals. Changes in laser power during acquisition of fluorescence images and image processing prior to reconstruction when using 2PLSM/FI may have affected reconstructed process diameter. Comparison of reconstructions based on FI or biocytin histology (BH) revealed a significant trend for those created using 2PLSM/FI to have larger process diameter than those based on BH (Figure 4).

We compared differences in average process diameter between the two reconstruction methods using L-measure. Diameter was consistently significantly larger for reconstructions made using FI for axonal and dendritic compartments of both cell types (Figure 4B). Differences in process diameter between reconstruction methods were investigated in more detail by comparing the diameter of many individually matched compartments for each PC dendrite using manual measurements (Figures 4C,D). All but one of the matched segments had a larger diameter when reconstructed from 2PLSM stacks (*n* = 25; *n* = 5 cells; FI vs. BH, 1.80 ± 0.15 μm vs. 0.91 ± 0.09 μm; *p* < 0.001). Taken together, these results show that FI reconstructions consistently exaggerate compartment diameter, on average and also typically for individual compartments.

### Effect of reconstruction method on single-cell modeling

**Figure 5:**
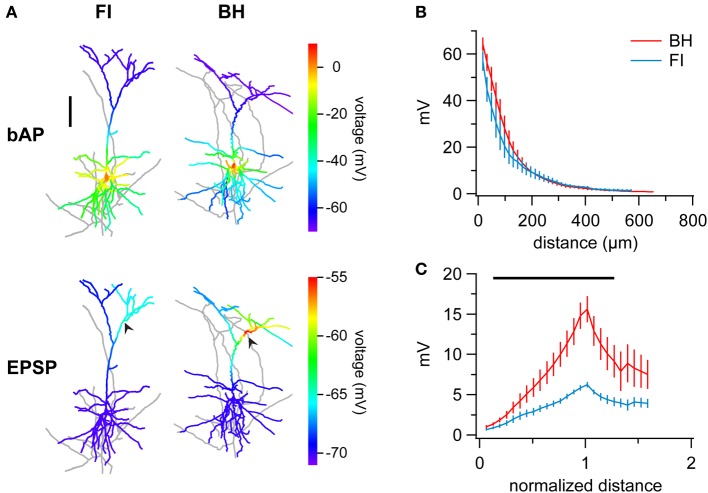
**FI reconstructions introduce errors in multicompartmental computer models. (A)** Sample reconstructions of the same cell indicating peak potentials resulting from of simulated back-propagating action potentials (bAPs) (top row) or forward-propagating EPSPs (bottom row). FI and BH reconstructions are on the left and right, respectively. Whilst bAP simulations are relatively similar, EPSP simulation results in smaller depolarization and local differences for the FI reconstruction. Arrows indicate the location of simulated synapses. Distal branches of morphologies are slightly cropped for clarity. **(B)** Ensemble averages of bAPs in PCs reconstructed using FI or BH, measured as peak amplitude at a given distance from the origin of the apical dendrite at the soma. Peak voltages were indistinguishable between methods at all distances. Vertical bars denote ± SEM. **(C)** Distance-normalized ensemble average of simulated forward-propagating EPSP amplitude in PCs reconstructed with either method shows a striking reduction of depolarizations in FI reconstructions. Distance from soma is normalized to the distance of the simulated synapse. Region of significance is indicated by black bar (paired *t*-test, *p* < 0.05).

A major use of 3D reconstructions of neurons is in single-cell and network modeling, using software such as NEURON (For review, see Brette et al., 2007). Differences between reconstruction methods, particularly in features such as process diameter, are expected to have considerable effects on the results of such modeling (Vetter et al., 2001; Tsay and Yuste, 2002; Acker and White, 2007). Complete morphological reconstruction may be vital for accurate simulation of features such as PC coincidence detection (Schaefer et al., 2003) or responses to stimulation such as whisker deflection (Sarid et al., 2013). To quantify these effects, we examined the effect of reconstruction method choice on single-cell modeling of action potential backpropagation (bAP) and EPSP forward propagation in the NEURON simulation environment (Figure 5), comparing models of the same cells based on morphologies generated using either BH or FI.

To investigate bAP simulations, we generated a rheobase spike at the soma of each model and recorded the resulting peak potentials in the apical dendrite at given distances away from the soma (Methods, Figures 5A,B). Interestingly, whilst models based on FI reconstructions exhibited a small trend for smaller depolarizations, this was indistinguishable between methods at all locations (Figure 5B). The effect of reconstruction method on modeling may thus be subtle and dependent on which aspects one is investigating. We should also point out that these findings might depend on the choice of model parameters; modifying the degree of dendritic excitability, for example, is not unlikely to bring out other differences.

Next, we investigated simulation of EPSP forward propagation. Here, we generated simulated EPSPs using the same parameters (see Methods) at matched locations on FI and BH reconstructions of the same cells, and measured resulting peak depolarizations across the morphology. Ensemble averaging of results revealed that simulations in FI reconstructions yielded smaller depolarizations (Figure 5C; areas where *p* < 0.05 indicated by bar). As EPSPs were generated at different distances from the soma in different cells, normalization of results to the somato-synaptic distance revealed the differences better, with FI reconstructions generating considerably smaller depolarizations (peak potential; BH vs. FI; 15.65 ± 1.63 mV vs. 6.27 ± 0.33 mV; *p* < 0.01; other areas of significance where *p* < 0.05 indicated by black bar in Figure 5C).

As systematic differences in process diameter may be expected to affect the spatial rate of voltage decay for both bAPs and EPSPs (Segev, 1998), we measured the length constant in each reconstruction (see Methods) and compared this between BH and FI. Surprisingly, the length constant did not vary significantly between methods (λ_BH_ = 308.518 ± 46.319 μm vs. λ_FI_ = 321.128 ± 65.185 μm, *p* = 0.80), despite FI systematically overestimating process diameters (see above). Presumably, this was because of other non-systematic differences between reconstruction methods and general variability that overshadowed the effect of diameter on length constant.

Overall, whilst differences in simulated bAPs were marked but not systematically different, there was a dramatic and consistent difference between methods in EPSP simulation, with FI reconstructions exhibiting smaller depolarizations in response to the same simulated synaptic stimulation. We therefore conclude that FI reconstructions are generally not suitable for multicompartmental computer modeling.

## Discussion

In this paper, we have quantified the effect of reconstruction method choice on morphometry and computer modeling by direct comparison of cells reconstructed using two commonly used methods. The one method, BH, is well established since many years back and is widely considered state of the art, for several good reasons. The other method, FI, is rapidly gaining in popularity, which is why it is important to know its pitfalls as well as its advantages in comparison to BH. By comparing these two methods, we have identified strengths and limitations of either method for such purposes, and we can in turn make recommendations as to the suitability of each for different applications. According to our results, FI is as a rule of thumb preferable for cell-type classification scenarios, whilst BH is superior for multicompartmental modeling and other applications requiring highly detailed tracing of thin arborizations with accurate diameter measurements.

### Quantitative morphological analysis and cell-type classification

One of the most common uses of 3D reconstructions such as those compared here is analysis of morphology, particularly in order to establish cell type. For example, axonal morphology is often cited as the most important determinant of cortical IN cell type (Markram et al., 2004; Wang et al., 2004; Toledo-Rodriguez et al., 2005; Ascoli et al., 2008). Increasingly, many properties of neural circuits such as synapse type and ion channel expression are found to be dependent on anatomical cell class (Blackman et al., 2013); therefore it is vital to accurately verify morphological type in any study where there may be cell-type-specific differences.

Our results indicate that FI and BH reconstructions are equal in providing an accurate representation of local morphology, with most morphological measures being indistinguishable between the two (Table 1). Unsupervised clustering results in successful separation of cell type in both methods (Figure 3). Whilst both methods appear to generate equivalent results for this purpose, FI reconstructions may confer a number of benefits that make them preferable in cell classification. Firstly, FI reconstructions, due to the ability to monitor FI online during electrophysiology experiments, effectively have a 100% yield for most purposes, as compared to the 50-80% yield of BH in our hands, which is dependent on post-recording histology (Figure 1). The lower yield of BH is highly dependent on the experimenter's experience and training with this state-of-the-art method, as well as on other factors such as cell type and age of the brain tissue. Although the yield can clearly be improved with experience and training, it will never reach 100%. FI-based reconstructions, however, are in our hands quite straightforward and are in fact an excellent training opportunity for volunteering undergraduate students who are just starting working in a lab. In addition, with FI, cell type may also be subjectively identified online whilst recording, increasing the throughput of electrophysiology experiments targeting a particular cell type. Furthermore, the unwanted distortions and shrinkage seen with BH reconstructions are avoided when using FI.

With all methodological comparisons, it is important to consider the costs involved. As FI reconstructions do not require histological processing or a dedicated setup for reconstruction, and image stacks can be acquired at the same time as electrophysiological recording, the time to generate a single reconstruction is much less than with BH, which can translate into saving running costs. Furthermore, FI reconstructions require less auxiliary equipment and use of consumables than BH reconstructions, resulting in lower cost per reconstruction. FI reconstructions do, however, require the initial high setup cost of the laser-scanning microscope, so this reasoning only applies for labs that already have access to 2PLSM or to confocal imaging. In our eyes, these benefits, together with the almost equal performance of FI and BH in revealing local morphology, make FI the preferred method in studies focusing on cell-type classification. This said, some cell types may extend over much larger areas than those described here (Lichtman and Denk, 2011). Whilst increasing fluorophore concentration, fill time and area imaged may increase the visible extent of FI reconstructions (see Figure 2), our results show that BH reconstructions reveal more distal processes (Table 1; e.g., hull width, max. Sholl radius, etc.), and therefore may be preferable if reconstruction over large distances is required. Even so, FI of axonal arborizations ranging several millimeters has successfully been carried out (see for example Pressler and Strowbridge, 2006; Williams et al., 2007), suggesting that this problem is possible to overcome by fine-tuning the FI reconstruction method. Mapping connectivity on larger scales using FI may be possible with whole-brain methods such as serial two-photon tomography (Ragan et al., 2012; Osten and Margrie, 2013).

### Multicompartmental computer modeling

Another major use of 3D reconstructions is in single-cell multicompartmental modeling. In this application, accuracy is paramount; even subtle differences in morphology may have considerable effects on both passive and active properties of neurons and models (Segev et al., 1995; Vetter et al., 2001). For example, dendritic morphology is thought to play a key role in the level of coupling in cortical pyramidal cell coincidence detection (Schaefer et al., 2003). Our results reveal that differences in morphology resulting from reconstruction method choice alone have large and significant effects on simulation of EPSP propagation. FI reconstructions consistently exhibit much smaller depolarizations than BH reconstructions (Figure 5).

The major contributing factor to these results is likely the large differences in dendritic diameter obtained between the two methods. Differences in measured process diameter alone would affect models of e.g., synaptic efficacy (Holmes, 1989) and voltage attenuation (Stuart and Spruston, 1998). Our results show that FI reconstructions consistently and significantly have larger process diameters, both on average and for matched compartments. As both BH and FI methods allow visualization of spines and axonal varicosities, a lack of spine detection is unlikely to be the cause of the larger diameters seen in FI. This finding is not unexpected, however, since increasing the laser power during acquisition of 2PLSM fluorescence images typically results in an apparent thickening of dendrites and axons. Neurite diameters obtained with 2PLSM are also subjectively affected by brightness/contrast settings during the reconstruction procedure, with a tendency for broadening of diameters when adjusting look-up tables to compensate for weak fluorescence. This problem seems much smaller with BH, presumably because the contrast produced with the histological amplification process is generally quite sufficient in and of itself. Due to the wavelength used, the theoretical resolution limit of light microscopes is also better than that of 2PLSM. This difference is compounded by the typical usage of high numerical aperture oil-immersion objectives with BH.

Although we have not tested this, we suspect that the neurite thickening problem might be considerably smaller with confocal microscopy than with 2PLSM, since its resolution limit is much better. It would be interesting to see a side-by-side comparison of FI reconstructions from 2PLSM and confocal microscopy stacks.

As diameter appears to be the main contributing factor for differences in computer modeling between FI and BH reconstructions of the same cells, it may be possible to correct for this, assuming that the differences are systematic. Preliminary results using a correction factor determined from differences in diameter of matched compartments suggest that it is possible to recover EPSP amplitudes in FI reconstructions to the levels seen with BH by manipulating diameter alone (data not shown). However, whilst it may be possible to determine specific correction parameters for a particular setup and experimenter by directly comparing diameter differences, these parameters may not be the same in alternate situations. For example, wide inter-experimenter differences in diameter and simulation results have been described when reconstructing from multiphoton data (Losavio et al., 2008). Another important factor to consider is that without technically demanding dendritic recordings, it is difficult to ascertain completely the ground truth, i.e., which of BH or FI is closer to reality. This said, the higher resolution and better signal-to-noise ratio found with BH justifies its position as a gold standard and as such BH reconstructions can be considered a benchmark or gold standard.

Because of the factors described above, and the large differences between EPSP modeling with FI and BH reconstructions, we recommend the use of BH in all multicompartmental modeling applications. This is further supported by the greater morphological detail revealed in BH reconstructions; it has been shown that even small differences in dendritic arborization may have large effects on the physiological properties of pyramidal cells (Schaefer et al., 2003), and simulations of such properties should therefore be based on the most accurate and complete morphological reconstructions possible. In contrast to neurite diameters and number of branches, the distortions and shrinkage seen with BH reconstructions are not likely to affect simulations much, and are therefore less of an issue for modeling as opposed to in morphometric applications (Schaefer et al., 2003). Until resolution-limit breaking FI reconstruction methods (see below) become commonplace, BH-based reconstructions are likely to remain state of the art for all multicompartmental computer-modeling applications.

### Alternative approaches and improvements

In this study we have chosen to focus on two commonly used methods to reconstruct detailed morphologies of single neurons, in order to provide a broadly applicable comparison of their strengths and weaknesses. However, a range of alternative methods are becoming increasingly available which may offer means to address some of the problems identified here, although these are often far more expensive, technically demanding and time-consuming.

For FI reconstructions, a key issue identified in this study is a potential lack of accuracy at levels of high detail, due to scattering of laser light in brain tissue, effects of image processing and a worse resolution limit than light microscopy. FI under the diffraction limit is however possible with super-resolution techniques such as structured illumination microscopy (SIM) or stimulated emission depletion (STED) (Hell, 2007; Ding et al., 2009; Evanko, 2009) and such methods potentially offer the ability to produce reconstructions at a detail suitable for accurate NEURON modeling using 2PLSM, although this would incur higher costs. An alternative way to create highly detailed reconstructions from FI is to use microinjection of fluorescent dyes in fixed tissue followed by confocal microscopy with deconvolution, although with this method anatomy cannot be combined with electrophysiology (Dumitriu et al., 2011). As noted above, confocal FI imaging may in general produce reconstructions with different properties to the 2PLSM derived reconstructions used here.

In contrast, a potential shortcoming of BH reconstructions identified in this study is the propensity to be affected by tissue distortions and deformations, particularly in the z-axis. Furthermore, there is a risk with BH of reconstructing from incompletely processed tissue—especially when a novice is first learning to use the technique—which may skew results. Recently, an improved biocytin staining protocol with slow dehydration and using the embedding medium Eukitt has been shared, which preserves some cytoarchitectonic features and allows for easier shrinkage correction in all dimensions (Marx et al., 2012). Compared with the far more common method used here, this may result in more realistic morphologies and allow for layer and area-specific morphometry without the use of markers such as cytochrome c oxidase. This method would also presumably result in even more accurate morphologies to be used in NEURON modeling. This said, it is not currently widely used and requires many more reagents than the standard protocol used in this study.

## Concluding remarks

In this study, we have quantitatively compared reconstructions from two popular methods (FI and BH) and identified consistent and significant differences in aspects of their resulting morphologies and use in computer modeling. Whilst both methods perform similarly for many morphological applications including cell classification, BH reconstructions reveal more distal neurites but suffer from compression and distortion artifacts. In computer modeling, FI reconstructions result in smaller simulated EPSPs, primarily due to the systematically larger diameters of cells reconstructed with this method. Therefore, care must be taken in reconstruction method choice for a particular application. In modeling studies particularly, mixing reconstructions from different methods may introduce measureable differences that do not represent that of underlying physiology and anatomy. In our hands, BH reconstructions are the gold standard for accuracy—however FI reconstructions are preferable for cell classification applications due to lower cost, higher throughput, and ease of use.

## Author contributions

Arne V. Blackman carried out experiments and morphometry. Stefan Grabuschnig did NEURON simulations. All authors contributed to analysis. Arne V. Blackman wrote the manuscript with input from co-authors.

### Conflict of interest statement

We would like to declare financial and infrastructure support from Scientifica that was provided as part of a BBSRC Industrial CASE Studentship (BB/H016600/1 - “*Using Novel Technology to Elucidate Neocortical Microcircuits with Multiple Simultaneous Whole-Cell Recordings*”). Scientifica co-funded Arne V. Blackman's stipend by £11,100.00 over 3 years and also provided the Sjöström lab with a combined 2-photon imaging and whole-cell recording rig for free over 4 years.
